# Novel C_H_1:C_L_ interfaces that enhance correct light chain pairing in heterodimeric bispecific antibodies

**DOI:** 10.1093/protein/gzx044

**Published:** 2017-08-31

**Authors:** Maximilian Bönisch, Carolin Sellmann, Daniel Maresch, Claudia Halbig, Stefan Becker, Lars Toleikis, Björn Hock, Florian Rüker

**Affiliations:** 1 Christian Doppler Laboratory for Antibody Engineering at Department of Chemistry and Department of Biotechnology, BOKU—University of Natural Resources and Life Sciences, Vienna, Muthgasse 18, A-1190 Vienna, Austria; 2 Institute for Organic Chemistry and Biochemistry, Technische Universität Darmstadt, Alarich-Weiss-Straße 4, D-64287 Darmstadt, Germany; 3 Department of Chemistry, BOKU—University of Natural Resources and Life Sciences, Vienna, Muthgasse 18, A-1190 Vienna, Austria; 4 Protein Engineering and Antibody Technologies, Merck KGaA, Frankfurter Straße 250, D-64293 Darmstadt, Germany

**Keywords:** bispecific antibodies, Fab interface design, heterodimeric IgG, light chain pairing problem, orthogonal Fab engineering

## Abstract

Targeting two unique antigens with a single bispecific antibody is an attractive approach with potential broad therapeutic applicability. However, the production of heterodimeric bispecific antibodies (bsAbs) presents a challenge, requiring the co-expression and accurate pairing of two distinct heavy and light chain units. Several undesirable by-products can be formed in the production process, including heavy chain homodimers and non-cognate light chain pairings. Although additional downstream purification methods exist, they are often time consuming and restrict practical large-scale production. In this study, we identify and validate novel Fab interface mutations that increase cognate light chain pairing efficiencies within heterodimeric bsAbs. Importantly, the variable domains remain unaltered as interface mutations were restricted to the C_H_1 and C_L_ domains. We performed several biochemical assays to demonstrate that the novel engineered interfaces do not adversely impact bispecific antibody expression, stability, affinity and biological function. The designs reported here can easily be applied in a generic manner to use existing antibodies as building blocks for bsAbs which will help to accelerate the identification and production of next generation bispecific antibody therapeutics.

## Introduction

Bispecific antibodies (bsAbs) target two unique epitopes on one or more antigen(s). BsAbs have several key advantages over monospecific antibodies. These advantages include the ability to recruit specific effector cells to cancer-associated epitopes, enhanced specificity through the dual recognition of cancer-associated antigens presented on a single cell, and the ability to simultaneously modulate two unique signalling pathways to limit cancer cell escape mechanisms (Krah *et al.*, 2017). To date, there are more than 50 bsAbs undergoing clinical trials, which highlights their recent gain in attention as promising clinical therapeutics (Brinkmann and Kontermann, 2017).

Maintaining the native IgG structure of a bsAb is favourable due to its well established properties as a therapeutic molecule, including the long *in vivo* half-life and the ability to elicit effector functions. However, the production of this type of bsAb remains technically challenging as light and heavy chain pairing can occur randomly. This results in the formation of several mispaired by-products (Schaefer *et al.*, 2015) and, ultimately, translates into a reduced yield.

A plethora of alternative formats based on immunoglobulin domains have been developed to produce bispecific molecules. Several of these formats avoid the complex assembly of a heterodimeric bispecific IgG (reviewed in Brinkmann and Kontermann, 2017). For example, the introduction of antigen binding sites within the C_H_3 domains of the Fc has been described which can be used for the production of homodimeric bsAbs (Wozniak-Knopp *et al.*, 2010). Other strategies require complex manufacturing steps and multiple cell lines (Strop *et al.*, 2012; Labrijn *et al.*, 2013; Spiess *et al.*, 2013). Although this has been shown to result in accurate pairing, the added manufacturing and assembly steps make this approach time consuming and industrially limiting. Hence, these major challenges in the production of bsAbs needed to be addressed: the heavy chain pairing problem and the light chain pairing problem.

The knobs-into-holes strategy was the first solution to the heavy chain pairing problem (Ridgway *et al.*, 1996; Atwell *et al.*, 1997). This approach focused on mutations within the C_H_3:C_H_3 interface, which promoted cognate heavy chain heterodimerisation. Since the identification of knobs-into-holes, several other groups have identified strategies based on interface engineering. While some rely on hydrophobic interactions (Von Kreudenstein *et al.*, 2013) others rely on electrostatic steering (Gunasekaran *et al.*, 2010). In contrast, the SEED technology is based on alternating segments of IgG and IgA C_H_3 sequences (Davis *et al.*, 2010). Although these strategies were successful in promoting cognate heavy chain pairing, they do not address the light chain pairing problem, which remains a considerable challenge.

**Fig. 1:**
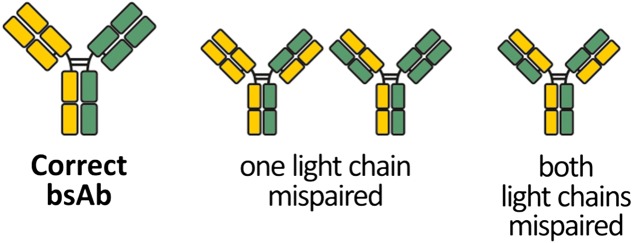
Chain pairing variants in bsAbs. A bsAb combines heavy and light chains of two different parent antibodies. The co-expression of all four chains in a single host cell potentially leads to various misassembled by-products. If heavy chain heterodimerisation is successful, three possible mispaired variants can still occur where one or two light chains bind to a non-cognate heavy chain.

When considering the light chain pairing problem, four possible pairing variants can be formed (Fig. 1), making the success rate of cognate light chain pairing ~25%. In recent years, progress has been made to limit the promiscuous pairing of the light chains, e.g. by domain cross over (Schaefer *et al.*, 2011). Others kept the native structure of the IgG and its arrangement of domains. For instance, an orthogonal Fab interface was created (Lewis *et al.*, 2014) or one interdomain disulphide bridge was rearranged (Mazor *et al.*, 2015) to direct both light chains to their cognate heavy chains. Furthermore, an electrostatic steering mechanism (Liu *et al.*, 2015) and electrostatic steering in combination with a knobs-into-holes approach (Dillon *et al.*, 2017) were reported to direct the light chains to their cognate heavy chains. Most of these designs include mutations in the V_H_:V_L_ interface which may adversely impact antigen binding.

In this study, we present novel Fab interface designs relying on mutations generated within the C_H_1:C_L_ interface. These mutations are shown to increase cognate light chain pairing efficiencies in heterodimeric bsAbs. The designs were developed using an *in silico* approach and extensive manual structure-guided screening. We demonstrate that these interface designs maintain high antibody expression yields and do not adversely impact thermal stability, antigen binding and biological function. Importantly, interface mutations are exclusively located within the constant region of the Fab and thus may be generically applicable to other bsAbs.

## Materials and Methods

### Alignments and modelling

Crystal structures 1oqo and 3eo9 were analysed using PyMOL (version 1.1r1). In 1oqo, residues 341–444 of chain A and residues 341–443 of chain B were copied to form one object representing the C_H_3:C_H_3 domains. In 3eo9, residues 122–219 of chain H and residues 108–213 of chain L were copied to form one object representing the C_H_1:C_L_ domains. These two objects were aligned with the align function (cycles = 50). To superimpose C_H_3 and C_L_, residues 341–443 of chain B of 1oqo were copied to form the C_H_3 object and residues 108–213 of chain L of 3eo9 were copied to form the C_L_ object. Objects C_H_3 and C_L_ were aligned using the align function (cycles = 50). The pair_fit function was used to focus the alignment of objects C_H_3 and C_L_ on the Cα atoms of residues 351, 366, 368, 395, 405, 407 and 409 of C_H_3 and residues 118, 133, 135, 163, 174, 176 and 178 of C_L_. Structures of interface designs were modelled with SWISS-MODEL (Arnold *et al.*, 2006; Benkert *et al.*, 2011; Biasini *et al.*, 2014) using the Fab fragment of trastuzumab (PDB ID: 4ioi) as template. Model structures were evaluated using MolProbity (Davis *et al.*, 2007; Chen *et al.*, 2010). Structure models were visualised using PyMOL. Sequence alignment of C_H_3, C_κ_ and C_H_1 domains of human IgG1 was obtained from the IMGT database (Lefranc *et al.*, 2015).

### Generation and production of 3D6^Q44E^ interface mutants

Mutations were introduced in pTT5 (National Research Council, Canada) containing anti-HIV gp41 antibody 3D6 (Grunow *et al.*, 1988; Felgenhauer *et al.*, 1990) heavy or kappa light chain coding sequences respectively using mutagenic primers and the QuikChange Lightning Site-Directed Mutagenesis Kit (Agilent Technologies, 210518) according to the manufacturer's protocol. The Q44E mutation was used previously to influence V_H_:V_L_ pairing (Igawa *et al.*, 2010). 3D6^Q44E^ and its interface mutants were produced by transient transfection of HEK293-6E cells (National Research Council, Canada) with two separate pTT5 plasmids for light and heavy chain at a molar ratio of 1:1. Cells were cultivated at 37°C in a 5% CO_2_ shaking incubator with humidified atmosphere in FreeStyle™ F17 Expression Medium (gibco, A1383501) supplemented with 4 mM l-Glutamine (gibco, 25030-024), 0.1% Pluronic F68 (gibco, 24040-032) and 50 μg/mL G-418 (gibco, 10131-027). Cells were transfected with 1 μg DNA per 1 mL culture using polyethylenimine (PEI, 25 kDa, linear from Polysciences, Inc., 23966). Two days post transfection (dpt), cells received Tryptone N1 (Organotechnie, 19553) at a final concentration of 0.5%. Five dpt the culture was spun down (300 g, 10 min) and the supernatant containing IgGs was filtered through a 0.45 μm nitrocellulose filter (Merck Millipore Ltd, HAWP04700). The supernatant was purified over a HiTrap Protein A HP column (GE Healthcare, 17-0402-01) using the lab-scale chromatography system ÄKTA purifier (GE Healthcare). Antibodies were eluted from the column by addition of 0.1 M glycine (pH 3.5). A 15 μL of 1 M TRIS (pH9) per 1 mL eluate was added to neutralise the pH. After dialysis against PBS, protein samples were analysed by analytical SEC with Superdex 200 10/300, running buffer PBS + 0.2 M NaCl, 50 μg of protein was loaded. Where indicated, IgGs were purified by preparative SEC using a Superdex 200 16/600 column.

### Production of bsAbs

Each of the four chains was present on a separate pTT5 plasmid. The molar ratio of plasmids during transfection was 2:1:1:1 (B10v5 heavy chain: B10v5 light chain: hu225M or huOKT3 heavy chain: hu225M or huOKT3 light chain). Production of bsAbs in HEK293-6E was performed as described above. Production of bsAbs in Expi293F™ was done using ExpiFectamine™ 293 Transfection kit (gibco, A14524) according to the manufacturer's instructions. Production of bsAbs in ExpiCHO-S was done using the ExpiCHO™ Expression System (gibco, A29133) following the instructions for the high titre protocol. Protein A purification, dialysis and SEC was done as described above.

### Analysis of 3D6^Q44E^ expression by western blot

5 dpt of HEK293-6E, crude supernatants were harvested and subjected to SDS-PAGE in reducing conditions (0.1 M DTT) using NuPAGE™ Novex™ 4–12% Bis-Tris Protein Gels (Invitrogen, NP0321BOX). Western blot was carried out using Peroxidase AffiniPure F(ab’)_2_ Fragment Goat Anti-Human IgG (H+L) (Jackson ImmunoResearch, 109-036-088) and Pierce™ CN/DAB Substrate Kit (ThermoScientific, 34000).

### IgG quantification by ELISA

Maxisorp plates were coated with anti-human IgG (Fc specific) fragment antibody (Sigma, I3391), 100 μL/well at 1 μg/mL in 1× PBS, pH 7.4, at 4°C overnight. After washing the plates three times with 1× PBS containing 0.1% Tween-20 (1× PBS-T), the plates were blocked using 2% BSA in 1× PBS for 1 h at room temperature. After washing the plates three times, 100 μL/well of crude cell culture supernatant in 1:3 serial dilutions was incubated for 1 h at room temperature. The plates were washed three times with 1× PBS-T and 100 μL/well anti-huFc-HRP (Sigma, A0170) diluted 1:6000 in 1× PBS-T was added. The plates were incubated at room temperature for 1 h, washed three times with 1× PBS-T and 100 μL/well TMB (Sigma, T0440) was added. The colorimetric reaction was stopped by addition of 100 μL/well of 30% H_2_SO_4_ and the absorbance of each well was determined at 450 nm and at 620 nm for reference.

### CD spectroscopy of C_L_ domains

Wt C_L_ and mutant C_L_ domains (residues 1.4–125, according to IMGT numbering, which is used throughout this report), all of kappa isotype, were expressed in HEK293-6E as described above. The C-terminal Cys was replaced by a GAA-linker sequence followed by a His_6_-tag. The cell culture supernatant was purified using a HisTrap HP column (GE Healthcare, 17-5247-01) equilibrated with buffer containing 20 mM NaPO_4_, 0.5 M NaCl and 20 mM imidazole. The C_L_ domains were eluted from the column by applying an imidazole gradient from 20 mM to 500 mM and dialysed against 1× PBS using a Slide-A-Lyzer™ Dialysis Cassette MWCO 2000 (ThermoFisher Scientific, 66203). The domains were analysed by electronic circular dichroism spectroscopy using Chirascan (Applied Photophysics, Leatherhead, UK). After flushing the instrument with nitrogen at a flow rate of 5 L min^−1^, the CD spectra (195–260 nm) for 350 μg/mL of protein solution were obtained. The path length was set to 1 mm, spectral band width was 3 nm and scan time per point 10 s. The data was analysed using Pro-Data Viewer (Applied Photophysics).

### Differential scanning calorimetry

Thermal stability was determined using a VP-capillary DSC microcalorimeter from Microcal (GE Healthcare) with a cell volume of 137 μl. C_L_ domains were measured at a concentration of 1 mg/mL and IgGs at 300 μg/mL in 1× PBS. The same buffer was used as a reference. The samples were measured from 20 to 100°C at a rate of 60°C h^−1^. The data was analysed using the software Origin 7.0. The buffer baselines were subtracted from the thermograms and the corrected thermograms were then normalised for protein concentration. The *T*_m_ values represent the peak maxima of the fitted curves.

### FoldX

Changes in stability and interaction energy caused by a mutation in the C_H_1:C_L_ interface were predicted using FoldX 3.0 Beta 6 (Guerois *et al.*, 2002; Schymkowitz *et al.*, 2005) in combination with the software YASARA (Version 13.9.8). The crystal structure of an IgG Fab with kappa light chain, PDB ID 3eo9 (Li *et al.*, 2009), was first modified by the <repairPDB> function. Next, a mutation was introduced using the <BuildModel> function. The resulting PDB file was used to mutate either of the two to three most adjacent residues on the opposite domain, exchanging it with all remaining amino acids except for cysteine. The FoldX algorithm then calculated the change in stability and the change in interaction energy as ΔΔ*G* values provided in kcal mol^−1^. Mutations resulting in ΔΔ*G* values below −0.5 kcal mol^−1^ were considered stabilising and were subsequently introduced in 3D6 by site-directed mutagenesis as described above.

### LC–ESI–MS

The protein A purified IgGs were digested with PNGase F (Roche) to release all *N*-glycans and were analysed using a Dionex Ultimate 3000 system directly linked to a QTOF instrument (maXis 4G ETD, Bruker) equipped with the standard ESI source in the positive ion mode. MS-scans were recorded within a range from 400 to 3800 *m*/*z*. Instrument calibration was performed using ESIcalibration mixture (Agilent). For separation of the proteins a Thermo ProSwift™ RP-4H Analytical separation column (250 × 0.200 mm^2^) was used. A gradient from 80% solvent A and 20% solvent B (Solvent A: 0.05% TFA, B: 80% ACCN and 20% solvent A) to 62.5% B in 15 min was applied, followed by a 5 min gradient from 62.5% B to 95% B, at a flow rate of 8 μL/min and 65°C. The analysis files were deconvoluted (Maximum Entropy Method, low mass: 40 000, high mass: 200 000, instrument resolv. power: 10 000) using DataAnalysis 4.0 (Bruker) and manually annotated. The maximum peak intensity of the deconvoluted spectra was taken to calculate the prevalence of each chain pairing variant relative to all detected heterodimeric IgGs. Traces of detected heavy chain homodimers were not included in the calculation. The prevalence of the variant with both light chains mispaired was estimated as described elsewhere (Yin *et al.*, 2016).

### Biolayer interferometry

Octet Red96 with Octet Data Acquisition software (v8.2, Forté Bio, Pall) was used to determine binding kinetics of bsAbs. Measurements were performed in 200 μL/well kinetics buffer (KB: PBS pH 7.4, 0.1% BSA, 0.02% Tween-20, Merck) at 30°C with 1000 rpm orbital agitation using black 96-well microplates (Greiner Bio One). Overall, 5 μg/mL antibodies diluted in DPBS (Life Technologies) were immobilised on anti-human IgG Fc capture biosensor tips (Forté Bio, Pall) pre-equilibrated in DPBS for 30 s. Next, biosensors were dipped in KB for 120 s to record a baseline. EGFR ECD or cMET ECD (produced in-house) served as analytes which were associated and dissociated for 600 and 1200 s, respectively. After subtracting the buffer controls, binding kinetics were calculated applying a global fitting algorithm and 1:1 Langmuir binding model.

Simultaneous binding was determined using streptavidin biosensor tips (Forté Bio, Pall). cMET ECD was biotinylated using EZ-LinkTM Sulfo-NHS-Biotinylation Kit (Thermo Scientific) and immobilised for 40 s at 5 μg/mL. The biosensors were then blocked for 60 s with 1% skimmed milk powder, 1% BSA, 0.1% Tween® 20 and 10 μg/mL biocytin. After incubation in KB for 120 s, the biosensors were first subjected to 50 nM bsAbs and then 50 nM EGFR ECD for 300 s each. As controls, each step was replaced by incubation in KB to exclude unspecific binding. Furthermore, constructs with only a single Fab (‘one-armed’) of either B10v5 (‘oa-B10v5’) or hu225M (‘oa-hu225M’) were produced, purified by SEC and analysed as described above.

### Western analysis of receptor phosphorylation

A549 cells were seeded in a 24 well plate at a concentration of 10^5^ cells/well and cultivated in DMEM + 10% FCS + 2 mM Na-Pyr. The day after, the media was replaced by starvation media (no FCS). The following day, cells received 300 nM of bsAbs or controls in starvation media for 3 h and were then stimulated with 100 ng/mL EGF and 100 ng/mL HGF (R&D Systems) for 10 min or left unstimulated. The cells were lysed with 50 μL/well RIPA buffer (Cell Signaling Technologies) supplemented with protease inhibitor (calbiochem, 539134) and phosphatase inhibitor (calbiochem, 524625) for 1 h on ice. Cell debris was spun down and protein concentration of the supernatant was determined using Bradford assay (Pierce Coomassie Plus). Equal amounts of total protein were loaded on an SDS-PAGE gel with subsequent immunoblotting using the following antibodies: rabbit anti-GAPDH (Cell signaling technology, 2118S), rabbit anti-cMET (US Biological, M3007-13A), rabbit anti-phospho-cMET (Cell signaling technology, 3077S), rabbit anti-EGFR (Cell signaling technology, 4267S), rabbit anti-phospho-EGFR (abcam, ab32578) and goat anti-rabbit IgG (H + L) alexa fluor®680 conjugate (life technologies, A21076). Blot imaging was done with LI-COR immunoblotting system (LI-COR).

## Results

### Rational design of candidate mutations

We employed a rational design approach to identify candidate mutations that could promote cognate light chain pairing within a heterodimeric bsAb. We specifically focused on the C_H_1 and C_L_ constant domains of the Fab as their locations far from the complementarity determining regions (CDRs) make mutations within these domains unlikely to adversely impact antigen binding. Candidate mutations were selected on two specific criteria; the mutations should disrupt pairing with a wildtype antibody chain and enhance pairing to a cognate engineered antibody chain.

**Fig. 2:**
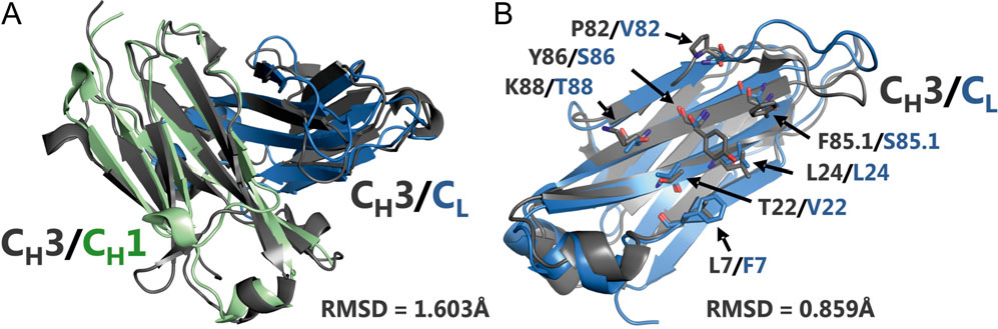
Structural alignments using the align function of PyMOL. (**A**) Superimposition of C_H_3:C_H_3 domains (PDB ID:1oqo) and the C_H_1:C_L_ domains (PDB ID: 3eo9). (**B**) Superimposition of C_H_3 and C_L_ as isolated objects. Interface residues in C_H_3 previously chosen to induce C_H_3:C_H_3 repulsion (Ying *et al.*, 2012) and their homologs in C_L_ are indicated.

We first sought to identify a strategy that would disrupt wildtype light chain–heavy chain interactions. Other groups have shown that specific interface mutations introduced within the C_H_3 domain can induce C_H_3:C_H_3 repulsion (Ying *et al.*, 2012). These published C_H_3 mutations included L7S, T22R, L24H, P82K, F85.1E, Y86K and K88A (IMGT numbering, see Table I-S for Eu and Kabat numbering). The align function of the software PyMOL was employed to superimpose crystal structures of the C_H_3:C_H_3 domains (PDB ID:1oqo) and the C_H_1:C_L_ domains (PDB ID:3eo9), demonstrating the high structural similarity of these domain pairs with an RMSD of 1.603 Å (Fig. 2A). We hypothesised that the repulsive effect produced by the residue mutations within the C_H_3 domain would be conserved within the C_H_1:C_L_ interface. Accordingly, sequences of C_H_3 and C_L_ were aligned and residues F7, V22, L24, V82, S85.1, S86 and T88 within the C_L_ domain were found to be homologous to the mutated positions in C_H_3 (Fig. 1-S). Structural alignment of C_H_3 and C_L_ alone revealed an RMSD of 0.859 Å (Fig. 2B). Furthermore, we focused the structural alignment on the seven aforementioned residues on both domains using the pair_fit function of PyMOL and obtained an RMSD of 0.882 Å. Based on this remarkably high degree of structural similarity of these two domain pairs we reasoned that the seven repulsive mutations originating from the C_H_3:C_H_3 interface could provide valuable guidance for engineering the C_H_1:C_L_ interface.

### Identifying and characterising repulsive C_L_ mutations within the monospecific 3D6^Q44E^ model antibody

**Fig. 3:**
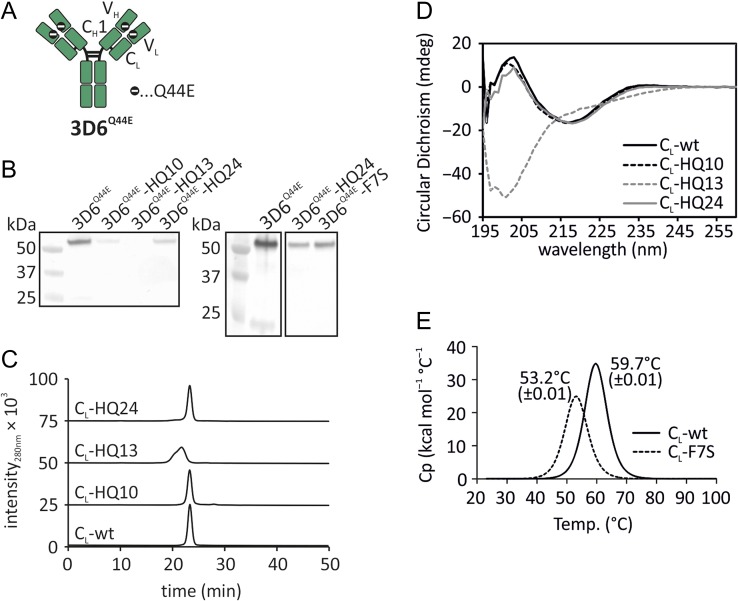
Identification of candidate mutants in C_L_. (**A**) Schematic drawing of the model antibody 3D6^Q44E^. The mutation Q44E was introduced in V_H_ and V_L_ to enhance the repulsive effect generated by mutations in the C_H_1:C_L_ interface. (**B**) Western blots to assess the impact of mutations in C_L_ on soluble antibody expression in HEK293-6E. Left, 3D6^Q44E^-HQ10 (S85.1E:S86K:T88A), 3D6^Q44E^-HQ13 (F7S:V22R:L24H:V82K:S85.1E:S86K:T88A) and 3D6^Q44E^-HQ24 (F7S:V82K). Right, effect on antibody expression of 3D6^Q44E^-HQ24 compared to single mutation F7S. (**C**) Analytical SEC and (**D**) circular dichroism of C_L_ domains with or without mutated interface residues. (**E**) Differential scanning calorimetry of wildtype C_L_ and C_L_-F7S.

As the first step of the engineering process, the monospecific 3D6^Q44E^ IgG antibody was employed in order to identify potentially repulsive mutations within the C_L_ domain that would disrupt light chain–heavy chain pairing interactions. The 3D6^Q44E^ model (Fig. 3A) is ideally suited for this study as it possesses destabilising mutations (Q44E, Igawa *et al.*, 2010) within the V_H_ and V_L_ domains, enhancing the repulsive effect generated by candidate C_H_1:C_L_ interface mutations, and thus greatly facilitating the engineering process.

Following the visual inspection of the C_H_3-derived candidate mutations, three constructs based on the 3D6^Q44E^ antibody were generated, containing the following C_L_ domain mutations: 3D6^Q44E^-HQ10 (S85.1E:S86K:T88A), 3D6^Q44E^-HQ13 (F7S:V22R:L24H:V82K:S85.1E:S86K:T88A) and 3D6^Q44E^-HQ24 (F7S:V82K). Previous studies (Feige *et al.*, 2009) suggested that mutations which impede light and heavy chain pairing would lead to decreased antibody expression. These reduced expression levels can serve as a surrogate for chain mispairing. Accordingly, Western blot analysis was performed and mutant IgG expression levels were compared to the parental 3D6^Q44E^ control (Fig. 3B, left). As predicted, all three mutants showed lower expression levels when compared to control. The HQ13 mutations, which incorporated all seven candidate mutations, resulted in the lowest expression level. When examining the structure of the HQ24 model, it was determined that the V82K side chain was directed away from the C_H_1:C_L_ interface and was unlikely to play a key role in chain pairing (data not shown). Accordingly, this mutation was omitted from the final design and was experimentally shown to be dispensable for the generation of reduced expression (Fig. 3B, right).

### Characterising biophysical properties within isolated mutant C_L_ domains

We performed size-exclusion chromatography (SEC) and circular dichroism (CD) to exclude the possibility that novel interface mutations could compromise the structure of the C_L_ domain. SEC revealed that the C_L_-HQ10 and C_L_-HQ24 models exhibited similar retention times and peak profiles when compared to the wildtype control (Fig. 3C). Circular dichroism revealed that the C_L_-HQ10 and C_L_-HQ24 models retained wildtype secondary structure profiles (Fig. 3D). In contrast, the C_L_-HQ13 showed aberrant SEC and circular dichroism profiles and was therefore excluded from further analysis (Fig. 3C and D).

An F7S mutation within the 3D6^Q44E^-F7S model was identified as producing a strong putative repulsive effect towards the wildtype C_H_1 domain. In addition, this model contained the fewest mutated residues and was therefore predicted to result in the lowest impact on domain structure. Based on these favourable findings, we focused on further validating the C_L_-F7S model via differential scanning calorimetry (DSC). DSC revealed a 6.5°C decrease in thermostability when compared to the C_L_-wt control (Fig. 3E). In summary, the C_L_-F7S model was revealed to produce adequate C_H_1 repulsion while maintaining acceptable thermostability and structural integrity. Accordingly, the C_L_-F7S model was selected as the lead repulsive mutation candidate.

### Identification of compensatory mutations in the C_H_1 domain

Following the identification and characterisation of repulsive C_L_ mutations within the 3D6^Q44E^ model, we next sought to identify compensatory mutations within the C_H_1 domain. Compensatory mutations were deemed as mutations that lead to increased expression compared to the 3D6^Q44E^ model containing a single F7S mutation. We hypothesised that residues located within the C_H_1 domain which were in close proximity to the F7S residue located on the C_L_ domain would possess the highest probability of restoring the C_H_1:C_L_–F7S interaction. Accordingly, residues L7, A20 and G22 in C_H_1 were deemed structurally proximal to the F7S mutation. The FoldX algorithm was employed to predict mutations producing a stabilising effect on the C_H_1:C_L_–F7S interaction. FoldX analysis failed to predict favourable stabilising interactions for residues L7 and G22. However, the A20L mutation was predicted to produce a favourable change in interaction energy (ΔΔ*G*) of −3.51 kcal mol^−1^ and a favourable change in stability of −2.08 kcal mol^−1^. Based on these promising energy predictions, the A20L mutation was selected for further *in vitro* analyses.

**Fig. 4:**
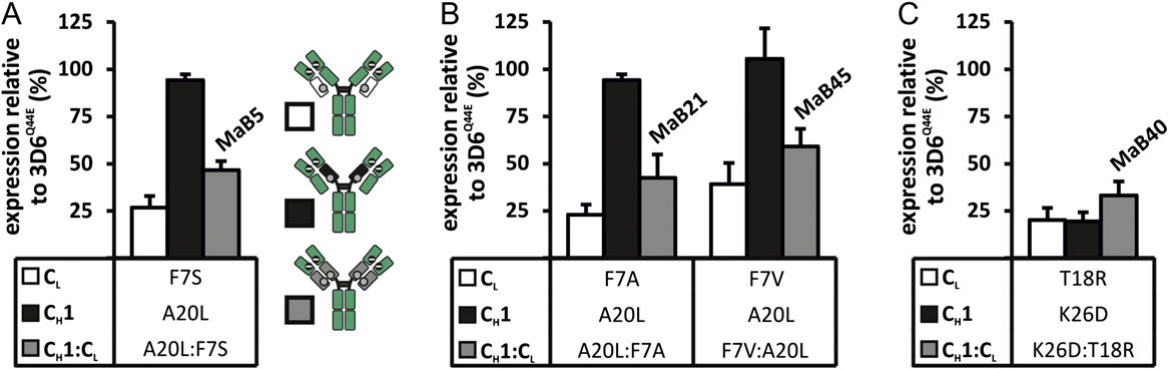
(**A**–**C**) Effect of mutations in C_L_ and C_H_1 on antibody expression. The antibody concentration in culture supernatants of HEK293-6E was determined 5 days post transfection using ELISA. 3D6^Q44E^ was expressed with one mutation in either C_L_ only (white), C_H_1 only (black) or in both C_L_ and C_H_1 (grey). The interface designs with mutations in both C_L_ and C_H_1 were named as indicated in the graphs. The expression is given relative to the parental antibody 3D6^Q44E^.

In order to test our above *in silico* prediction, the A20L mutation was incorporated into the C_H_1 domain of the 3D6^Q44E^ heavy chain and the A20L-containing heavy chain was co-expressed with the F7S-containing light chain, yielding an interface named MaB5. To evaluate expression levels, ELISA was employed to determine IgG concentration levels in culture supernatants. When comparing the expression level of MaB5 to the parental 3D6^Q44E^-F7S control, MaB5 showed a higher expression suggesting a restored C_H_1:C_L_ interaction (Fig. 4A). However, the expression of 3D6^Q44E^-A20L was comparable to the parental antibody 3D6^Q44E^ which indicated that A20L does not possess repulsive properties. When taken together, the MaB5 interface was deemed a promising candidate to enhance cognate light chain pairing. However, due to the inability of the A20L mutation to produce a repulsive effect towards a wildtype light chain, we broadened our search for additional candidate mutations.

### Discovery of additional interface mutations

After identifying a promising candidate, we next sought to further optimise the interface by evaluating alternative amino acid substitutions at position 7 within the C_L_ domain. Alanine and valine substitutions at position 7, when paired with the A20L mutation found on the C_H_1 domain, were identified as producing a similar effect as the F7S mutation (Fig. 4B). The A20L:F7A interface and the A20L:F7V interface were named MaB21 and MaB45, respectively.

To identify additional candidate mutations, FoldX was employed to evaluate compensatory mutations that restored the C_H_1 interaction with the 3D6^Q44E^-HQ10 light chain. Several compensatory mutations were identified but failed experimental validation. Interestingly, a K26D mutation on the C_H_1 domain that did not produce a compensatory effect, produced a putative repulsive effect. Based on this finding, we next sought to identify a mutation on the C_L_ domain that would restore the C_H_1:C_L_ interaction. FoldX predicted a T18R mutation within the C_L_ domain would restore the C_H_1:C_L_ interaction and this mutation was subsequently experimentally validated (Fig. 4C). Independently, the K26D mutation and the T18R mutation produce a putative repulsive effect when co-expressed with their respective wildtype binding partners. However, expression was higher when both mutations were present in the C_H_1:C_L_ interface, indicating interactions between both domains were restored. This novel engineered interface was designated MaB40 and was identified as an additional promising candidate that was evaluated in subsequent validation experiments.

**Fig. 5:**
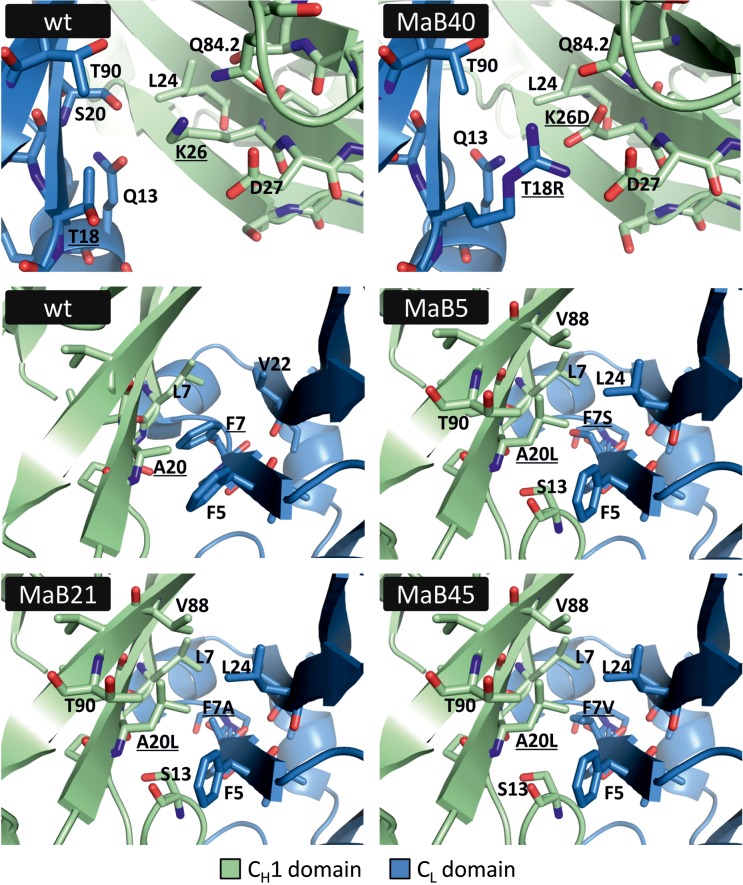
Interface designs MaB5, MaB21, MaB40, and MaB45 compared to the respective sections of the wildtype C_H_1:C_L_ interface (PDB ID: 3eo9). The structures of interface designs were modelled using SWISS-MODEL. Residues chosen for mutation in the wildtype structure and mutated residues in the structure models are underscored. Neighbouring residues within a radius of 4 Å are shown.

Taken together, four unique candidate interface designs were identified. In order to assess the changes introduced to the C_H_1:C_L_ interface, SWISS-MODEL was employed to obtain structural models. These model pdb files were subsequently analysed using MolProbity and were found to be of acceptable quality (data not shown). Possible side-chain contacts between the mutated amino acids were visually inspected using PyMOL (Fig. 5). The possibility for ionic interaction between mutated residues K26D and T18R is clearly indicated. Furthermore, the model structures suggest that mutations involving residues A20 within the C_H_1 domain and F7 within the C_L_ domain allow favourable interactions.

### The novel MaB40 interface increases cognate light chain pairing in a subset of established cell lines

We next investigated the impact of candidate interfaces on chain pairing in a model bsAb. We selected the B10v5 × hu225M bsAb as it has been previously well characterised (Sellmann *et al.*, 2016), contains light chains with dissimilar molecular weights which allows for accurate mass spectrometry analyses, and employs the SEED technology to ensure accurate heavy chain heterodimerisation. The B10v5 × hu225M bsAb targets the cMET antigen with its B10v5 Fab arm and the EGFR antigen with its hu225M scFv arm. To allow for light chain pairing testing, we reformatted the scFv arm to a fully functional Fab arm.

**Fig. 6:**
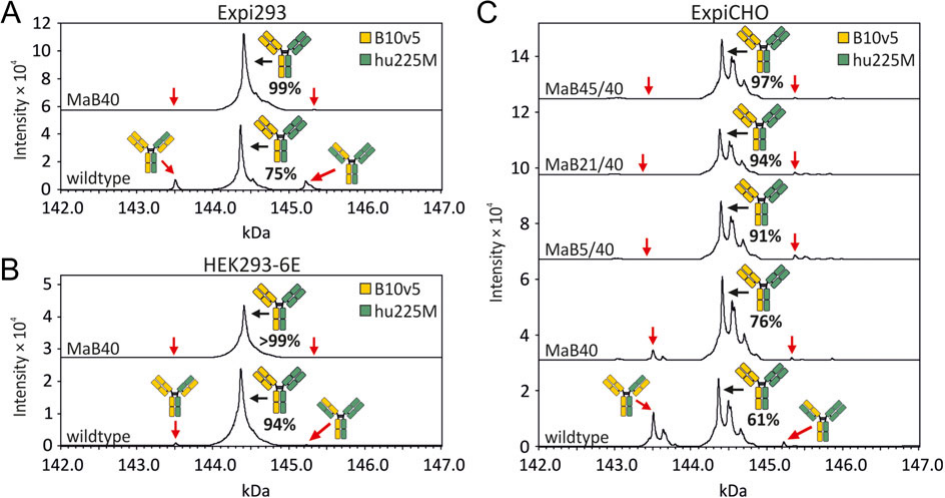
Analysis of pairing of light to heavy chain in the bsAb B10v5 × hu225M by LC–ESI–MS, after protein A purification and deglycosylation. The bsAbs contained either no mutation in the Fab interface (wildtype) or contained mutations in the C_H_1:C_L_ interface (see Table II for a complete list). The V_H_:V_L_ interface was left unmutated in all cases. The antibodies were produced by transient transfection of (**A**) Expi293, (**B**) HEK293-6E or (**C**) ExpiCHO. The prevalence of correctly assembled bsAb is given in percent of all detected heterodimeric IgGs and represents a mean of two (A) or three (B and C) independent transfections (Table I).

The MaB40 interface was incorporated into the hu225M Fab arm of the B10v5 × hu225M bsAb as it was deemed the most promising candidate. Notably, the Q44E mutations that were previously employed in interface design development were not incorporated in the bsAbs evaluated below. In order to evaluate if the novel interface design would result in increased light chain pairing accuracy, Expi293 cells were transfected with either the B10v5 × hu225M-MaB40 or the wildtype B10v5 × hu225M control. Pairing accuracies were evaluated via LC–ESI–MS and several peak profiles that could accurately differentiate between cognate and non-cognate pairing were observed (Fig. 6A and Table I). Specifically, mass spec revealed that wildtype B10v5 × hu225M bsAb expression within the Expi293 cell line resulted in ~75% cognate light chain pairing. As predicted, mass spec profiles of the B10v5 × hu225M-MaB40 bsAb revealed an increase in cognate pairing with ~99% of bsAb being accurately paired when expressed within the Expi293 cell line.

**Table I.** Prevalence of correctly assembled B10v5 × hu225M and mispaired variants in percent of all detected heterodimeric IgGs analysed by LC–ESI–MS. Values represent a mean of two (Expi293) or three (HEK293-6E and ExpiCHO) independent transfections ± standard deviation

Design	Correct bsAb (%)	One light chain mispaired (%)		Both light chains mispaired (%)^a^
Expi293				
wt	74.9 ± 1.0	10.6 ± 1.9	12.7 ± 1.0	1.8 ± 0.2
MaB40	98.8 ± 0.7	0.9 ± 1.2	0.4 ± 0.5	0.0 ± 0.0
HEK293-6E				
wt	93.6 ± 3.3	4.6 ± 1.7	1.6 ± 1.6	0.1 ± 0.1
MaB40	99.5 ± 0.9	0.5 ± 0.9	0.0 ± 0.0	0.0 ± 0.0
ExpiCHO				
wt	61.4 ± 5.2	34.6 ± 3.1	2.5 ± 2.2	1.5 ± 1.3
MaB40	76.3 ± 11.5	21.9 ± 11.7	1.4 ± 1.3	0.4 ± 0.4
MaB5/40	91.0 ± 3.7	0.0 ± 0.0	9.0 ± 3.7	0.0 ± 0.0
MaB21/40	94.1 ± 1.5	0.0 ± 0.0	5.9 ± 1.5	0.0 ± 0.0
MaB45/40	96.6 ± 2.8	0.0 ± 0.0	3.4 ± 2.8	0.0 ± 0.0

^a^Estimate calculated according to Yin *et al.* (2016).

**Table II.** Names of tested interface designs and their respective mutations in the bsAbs B10v5 × hu225M and B10v5 × huOKT3

Design	B10v5		hu225M or huOKT3	
	C_H_1	C_L_	C_H_1	C_L_
MaB40	–	–	K26D	T18R
MaB5/40	A20L	F7S	K26D	T18R
MaB21/40	A20L	F7A	K26D	T18R
MaB45/40	A20L	F7V	K26D	T18R

In order to evaluate if the observed effect on light chain pairing was conserved in other cell lines, we specifically tested our novel interface on two additional established cell lines commonly employed in antibody production. HEK293-6E cells were transfected with either the B10v5 × hu225M-MaB40 or the wildtype B10v5 × hu225M control. Pairing accuracies were evaluated via LC–ESI–MS (Fig. 6B, Table I). LC–ESI–MS revealed pairing accuracies of 94% and over 99% for the wildtype B10v5 × hu225M and B10v5 × hu225M-MaB40, respectively. Although it was again observed that the MaB40 interface design resulted in an increase in cognate pairing, a baseline increase in the cognate light chain pairing of the wildtype B10v5 × hu225M control was also observed.

We next sought to evaluate the novel interface expressed within the ExpiCHO cell line. Using an analogous approach as detailed above, the wildtype B10v5 × hu225M and B10v5 × hu225M-MaB40 models exhibited a cognate pairing efficiency of 61 and 76%, respectively (Fig. 6C and Table I). Notably, additional peaks were observed when examining the LC–ESI–MS data. After further analysis, it was hypothesised that additional peaks likely represented incomplete removal of C-terminal lysine residues and additional glycation events, two phenomena which are commonly observed in the production of IgGs (Liu *et al.*, 2008).

As only a modest increase in cognate paring was observed within the ExpiCHO cell line we hypothesised that additional mutations may potentially result in enhanced cognate pairing. To test this hypothesis, we incorporated mutations that were previously identified (MaB5, MaB21, MaB45) during the initial interface design protocol and evaluated if they could produce enhanced cognate pairing within the B10v5 Fab interface. Table II lists all tested bsAbs and their respective mutations. Remarkably, all three additional interface designs resulted in an increase in cognate light chain pairing with 91, 94 and 97% cognate pairing in the B10v5 × hu225M-MaB5/40, -MaB21/40 and -MaB45/40 bsAbs, respectively (Fig. 6C and Table I). In summary, interface mutations resulted in an increase in cognate light chain pairing efficiencies relative to controls. Interestingly, it was observed that cognate light chain pairing percentages varied across the cell lines tested.

### The novel MaB40, MaB5/40, MaB21/40 and MaB45/40 interfaces do not adversely impact the biophysical and functional properties of the B10v5 × hu225M bsAb

In order to evaluate the impact of the engineered interfaces, we performed several biophysical and functional tests. We first sought to evaluate the impact on antibody yield in a small scale (33 mL culture volume) antibody production assay. BsAbs were harvested from ExpiCHO cell culture supernatants, were protein A purified, and quantified spectrophotometrically. Protein yields were reported as maximums: 370 mg/L for the wildtype B10v5 × hu225M, 300 mg/L for B10v5 × hu225M-MaB40, 190 mg/L for -MaB5/40, 200 mg/L for -MaB21/40 and 210 mg/L for -MaB45/40.

**Fig. 7:**
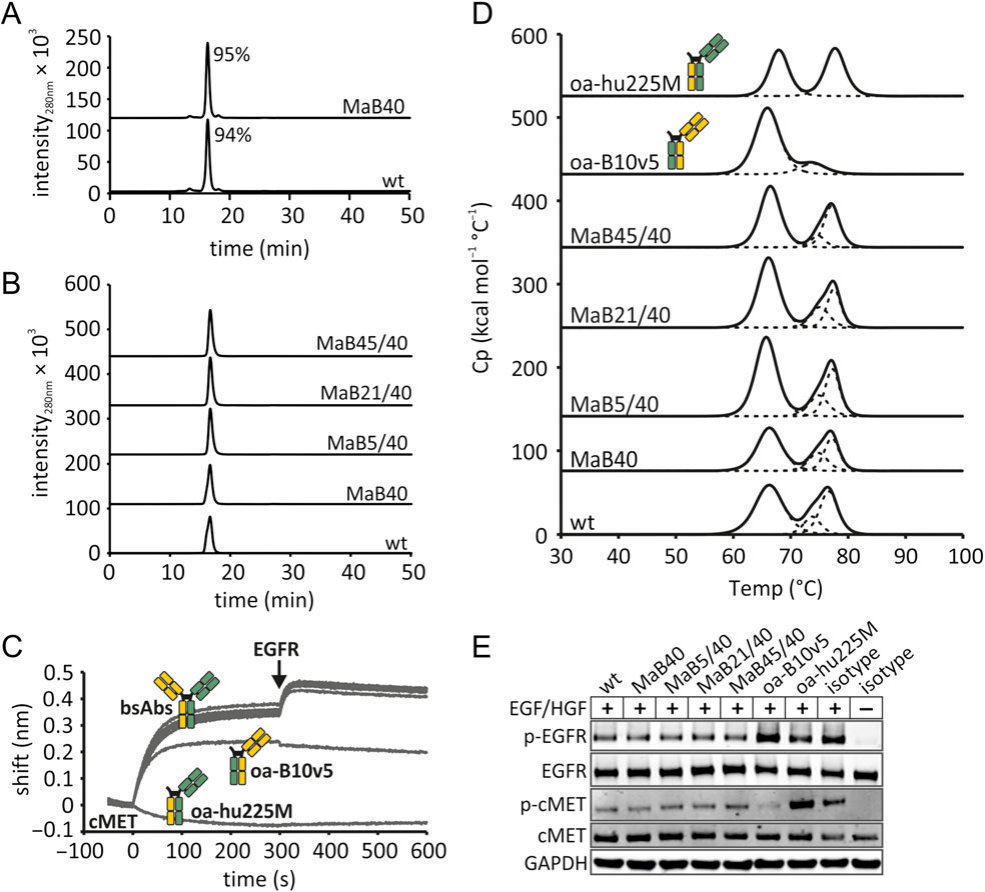
Biophysical and functional characterisation of B10v5 × hu225M with or without mutations in the C_H_1:C_L_ interface. Analytical SEC after protein A purification of bsAbs produced in (**A**) HEK293-6E or (**B**) bsAbs produced in ExpiCHO after preparative SEC. The area under the curve in percent of all detected peak areas is given in the graph. (**C**) Simultaneous binding of both antigens cMET and EGFR using biolayer interferometry. BsAbs (wt, MaB40, MaB5/40, MaB21/40, MaB45/40) or antibodies consisting of only one Fab (oa for ‘one-armed’) were allowed to bind to cMET coated biosensors. Subsequently, biosensors were incubated with EGFR. (**D**) Differential scanning calorimetry of wildtype, interface mutants and ‘one-armed’ constructs (Table III). (**E**) Western blot analysis of the inhibition of EGFR and cMET phosphorylation in A549 cells by bsAbs with our without interface designs.

Following Protein A purification from HEK293-6E supernatants, analytical SEC was performed on the B10v5 × hu225M-MaB40 bsAb. The SEC profile was predominantly monomeric and virtually indistinguishable from the wildtype B10v5 × hu225M control (Fig. 7A). The B10v5 × hu225M-MaB40, -MaB5/40, B10v5 × hu225M-MaB21/40, B10v5 × hu225M-MaB45/40 interface mutants and the wildtype B10v5 × hu225M control were produced in ExpiCHO and subjected to preparative SEC. Following purification, analytical SEC was performed and SEC profiles were predominantly monomeric and virtually indistinguishable from the wildtype B10v5 × hu225M control (Fig. 7B).

**Table III.** Affinity to both antigens and thermal stability of B10v5 × hu225M with different interface designs or ‘one-armed’ constructs as controls

Design	KD (nM)		*T*_m_ (°C)		
	cMET	EGFR	Peak 1	Peak 2	Peak 3
wt	0.4	5.0	66.3	73.7	76.6
MaB40	0.4	6.0	66.3	74.8	77.1
MaB5/40	0.5	2.9	65.8	74.9	77.3
MaB21/40	0.5	7.2	66.2	75.1	77.5
MaB45/40	0.4	4.0	66.5	74.8	77.1
oa-B105	0.3	n.d.	65.9	73.8	–
oa-hu225M	n.d.	6.3	67.9	–	77.7

*T*_m_, melting temperature; KD, equilibrium dissociation constant; n.d. not determined.

Biolayer interferometry was employed to verify that following interface engineering, dual antigen binding was retained. Biolayer interferometry with cMET coated biosensors revealed that the bsAbs (B10v5 × hu225M-MaB40, -MaB5/40, -MaB21/40, -MaB45/40 and the wildtype B10v5 × hu225M) bound to the cMET antigen and could subsequently bind the EGFR antigen. As expected, the one-armed constructs did not exhibit dual antigen binding (Fig. 7C). Affinities of the mutant bsAbs were similar to the wildtype, indicating that the interface engineering did not negatively impact antigen binding kinetics (Table III).

To determine the impact of the interface mutations on thermal stability, we performed DSC (Fig. 7D and Table III). The *T*_m_ of the SEED domains is reported to be 67.7°C (Muda *et al.*, 2011). The melting temperatures of both Fabs were determined by analysing the ‘one-armed’ constructs. The Fab of oa-hu225M exhibited a *T*_m_ of 77.7°C and the Fab of oa-B10v5 exhibited a *T*_m_ of 65.9°C, coinciding with the unfolding of the SEED domains. When examining the bsAbs with interface mutations the melting temperatures of both Fabs were similar to the ones observed in the wildtype. This demonstrates that the engineered Fab interfaces do not negatively impact thermal stability.

Next, we employed Western blot analyses and phosphorylation-specific antibodies to confirm that our engineered bsAbs retain their reported ability to inhibit EGFR and cMET phosphorylation in A549 cells (Sellmann *et al.*, 2016) (Fig. 7E). As expected, all engineered bsAbs and the wildtype control successfully reduced phosphorylation levels of the EGFR and cMET receptors.

### Increased cognate light chain pairing, resulting from engineered interfaces, is conserved in another bsAb

**Fig. 8:**
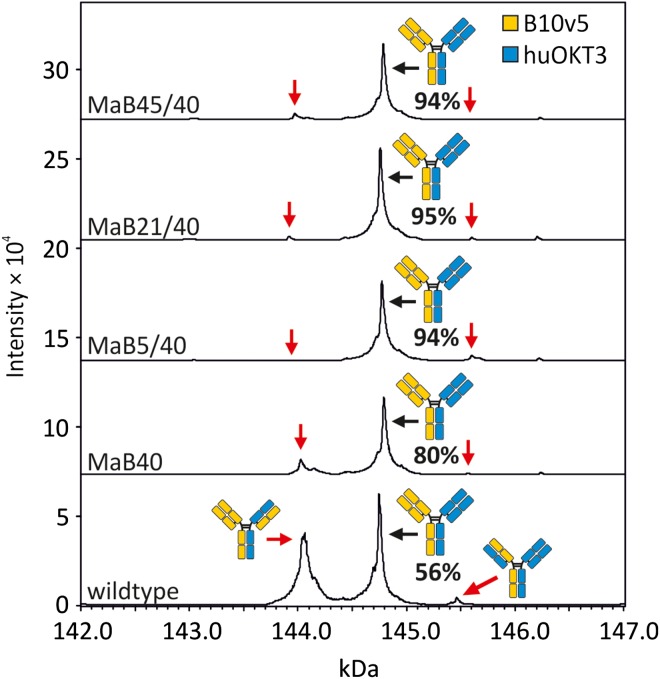
Analysis of pairing of light to heavy chain in the bsAb B10v5 × huOKT3 by LC–ESI–MS, after protein A purification and deglycosylation. The bsAbs contained either no mutation in the Fab interface (wildtype) or contained mutations in the C_H_1:C_L_ interface (see Table I for a complete list). The V_H_:V_L_ interface was left unmutated in all cases. The antibodies were produced by transient transfection of HEK293-6E. The prevalence of correctly assembled bsAb is given in percent of all detected heterodimeric IgGs and represents a mean of three independent transfections (Table IV).

We next sought to evaluate if the engineered interfaces could be employed in an additional bsAb. Accordingly, we combined the B10v5 antibody with the huOKT3 antibody (Table I) and evaluated cognate light chain pairing efficiencies via LC–ESI–MS. Compared to the 56% cognate pairing observed within the wildtype control, the four interface designs resulted in 80, 94, 95 and 94% cognate pairing in the B10v5 × huOKT3-MaB40, -MaB5/40, -MaB21/40 and -MaB45/40 bsAbs, respectively (Fig. 8 and Table IV). In summary, interface designs increased the percentage of cognate light chain pairing relative to controls.

**Table IV.** Prevalence of correctly assembled B10v5 × huOKT3 and mispaired variants in percent of all detected heterodimeric IgGs analysed by LC–ESI–MS. Values represent the mean of three independent transfections of HEK293-6E ± standard deviation

Design	Correct bsAb (%)	One light chain mispaired (%)		Both light chains mispaired (%)^a^
wt	55.7 ± 0.4	38.3 ± 6.3	3.8 ± 3.7	2.3 ± 2.2
MaB40	80.2 ± 5.1	19.0 ± 5.8	0.7 ± 0.6	0.1 ± 0.1
MaB5/40	93.5 ± 2.6	0.0 ± 0.0	6.5 ± 2.6	0.0 ± 0.0
MaB21/40	94.6 ± 2.7	2.8 ± 1.0	2.5 ± 1.9	0.1 ± 0.1
MaB45/40	93.8 ± 1.1	5.5 ± 1.1	0.7 ± 1.2	0.0 ± 0.1

^a^Estimate calculated according to Yin *et al.* (2016).

## Discussion

In this study, we employed a rational design approach to identify candidate mutations that could promote cognate light chain pairing within a heterodimeric bsAb. We describe novel mutations located solely within the C_H_1:C_L_ interface that were experimentally shown to enhance cognate pairing. We specifically identified mutations A20L and K26D within the C_H_1 domain and F7S, F7A, F7V and T18R within the C_L_ domain as serving important roles in generating a heterodimeric bsAb with cognate light chain pairing. Importantly, it was shown that mutating a minimal number of residues was sufficient to enhance cognate light chain pairing which minimises the possible impact of the engineering on antibody structure and function. Specifically, when examining the Expi293 cell line, we observed an increase in cognate light chain pairing from 75 to 99%. When examining the HEK293-6E cell line we observed an increase in cognate light chain pairing from 94 to over 99%. When examining the ExpiCHO cell line we observed an increase in cognate light chain pairing from 61 to 97%. Collectively, in all three cell lines tested, the novel interfaces were shown to enhance cognate light chain pairing. To demonstrate that interface mutations did not adversely impact bsAb structure or function, they were validated through several biophysical and functional tests. Interestingly, when examining the wildtype B10v5 × hu225M bsAb, we observed unexpected differences in cognate light chain pairing efficiencies across three distinct cell lines. Specifically, we observed a 75, 94 and 61% cognate light chain pairing efficiency in Expi293, HEK293-6E and ExpiCHO, respectively. In summary, within all three cell lines examined, novel interfaces enhanced cognate light chain pairing.

Our study shows that through a rational design approach, we identified novel C_H_1:C_L_ interfaces that were capable of increasing cognate pairing up to 99% without affecting bsAb structure and function (Figs 6–8). Indeed, other groups have demonstrated the ability to increase light chain pairing efficiencies through interface engineering (Lewis *et al.*, 2014; Liu *et al.*, 2015). For example, Lewis *et al.* mutated six residues within the variable domains and four residues within the constant domains to elicit increases in cognate light chain pairing. When they employed this interface design in HEK293F and in combination with anti-HER2 × anti-EGFR bsAb or anti-cMET × anti-Axl bsAb, they showed an increase of light chain pairing efficiencies from 65 to 93% and 64 to 90%, respectively. In comparison, we mutate up to two residues within the C_H_1 domains and up to two residues within the C_L_ domains to elicit enhanced cognate light chain pairing and achieve up to 97% pairing efficiency when expressed within ExpiCHO. There are several potential differences that could account for the discrepancies between these studies including differences between expression cell lines employed, differences between bsAbs that were created, and quantities of DNA transfected. Although both studies were capable of producing enhanced cognate light chain pairing efficiencies, our approach employs minimal interface mutations that are restricted to the constant domains and thus may mitigate any potential negative impact to the CDRs when employed in other bsAbs. Our findings are in agreement with other groups who show increased cognate light chain pairing efficiencies via interface mutations restricted to the constant domains (Mazor *et al.*, 2015; Dillon *et al.*, 2017).

Differences in light chain pairing efficiencies, in the absence of interface design, were observed between cells lines expressing an identical bsAb (Fig. 6). An identical molar ratio of each antibody chain plasmid was employed throughout this study to mitigate any confounding factors associated with a difference in chain ratios. When evaluating mispaired variants of B10v5 × hu225M in the absence of interface design, cognate light chain pairing was observed to be 75, 94 and 61% in Expi293, HEK293-6E and ExpiCHO, respectively.

Interestingly, the prevalence of cognate light chain pairing negatively correlated with bsAb total yield (data not shown). Accordingly, the highest percentage of cognate pairing was observed in HEK293-6E, the cell line that produced the lowest yield. On the other hand, the lowest percentage of cognate pairing was observed in ExpiCHO, the cell line that produced the highest yield. It is tempting to speculate that the over-expression of bsAbs within high expressing cell lines may overwhelm inherent quality control mechanisms and result in increased percentages of mispaired variants. Based on these observations, there appears to be a trade-off between high expression and cognate pairing. Accordingly, interface engineering may serve to minimise this trade-off by increasing cognate pairing within high expressing cell lines.

It is important to note that in the production of bsAbs, considerable non-cognate light chain pairing was not always observed. For example, in the absence of engineered interfaces, we detected 94% cognate light chain pairing of B10v5 × hu225M in HEK293-6E. These observations highlight the importance of evaluating cognate chain pairing efficiencies in the presence and absence of interface engineering. This allows for the characterisation of baseline cognate light chain pairing efficiencies and for determining the efficiency of one's interface design. Nevertheless, in the HEK293-6E and ExpiCHO cell lines, the interface designs described here enhanced cognate light chain pairing to over 99 and 97%, respectively (Fig. 6).

We observed 61% cognate light chain pairing in wildtype B10v5 × hu225M produced in ExpiCHO (Table II). Similarly, we observed 56% cognate light chain pairing in wildtype B10v5 × huOKT3 produced in HEK293-6E (Table IV). The prevalence of cognate light chain pairing is in agreement with other studies describing 60–70% cognate light chain pairing in the absence of interface designs (Lewis *et al.*, 2014; Dillon *et al.*, 2017). Since a prevalence of only 25% is statistically predicted, this may indicate an inherent preference for cognate light chain pairing under certain conditions. It remains to be tested if our interface designs are effective in bsAbs where light chain pairing is entirely random. Indeed, bsAbs have been described where no apparent preference for cognate light chain pairing has been observed (Schaefer *et al.*, 2015; Dillon *et al.*, 2017).

Several studies have begun to shed light on heavy and light chain pairing preferences. However, either no preference or only a limited preference for certain V_H_ and V_L_ families to recombine (Brezinschek *et al.*, 1998; Jayaram *et al.*, 2012) was found. Other groups have proposed that in particular the CDRs influence the pairing preferences of heavy and light chains (Dillon *et al.*, 2017). In conclusion, further investigations are required to understand the conditions under which a preference for cognate light chain pairing can occur.

To date, several groups have identified numerous interface pairing strategies. Many of these strategies have focused on increasing accurate heavy chain heterodimerisation. In this study, we employ C_H_1:C_L_ interface engineering in combination with the SEED technology to achieve correctly assembled bsAbs. However, the novel interface designs presented here are expected to be compatible with other heavy chain heterodimerisation strategies and accordingly have the potential to serve a universal role in bsAb design and production. During interface design development we utilised 3D6^Q44E^, an IgG antibody containing kappa light chains. However, enhanced cognate light chain pairing was also observed in B10v5 × hu225M where the designs MaB5, MaB21 and MaB45 were incorporated into the lambda light chain-containing B10v5 Fab. This further extends the applicability of these interface designs as they have the potential to function within both light chain isotypes.

In summary, we have identified C_H_1:C_L_ interfaces that promote efficient cognate light chain pairing in two heterodimeric bsAbs. Importantly, our strategy employs residues within the constant domains and thus mitigates any potential impact to the variable domains. The interface designs did not adversely affect antibody expression yields, thermal stability, antigen affinity, dual antigen binding capabilities and biological function. These novel interface designs may accelerate the identification and production of the next generation of lead bsAb therapeutics and may broadly serve as a general antibody engineering strategy.

## Supplementary Material

Supplementary Data
