# Supplementary material for: Novel CH1:CL interfaces that enhance correct light chain pairing in heterodimeric bispecific antibodies
Source: Protein Eng Des Sel. 2017 Aug 31;30(9):685–96. doi: 10.1093/protein/gzx044 (PMC5914326; doi:10.1093/protein/gzx044)
Supplement: Supplementary Data [file supplementary_material.doc]

| **Table I-S.** Residues covered during the rational design process and their respective numbers according to IMGT, Eu, and Kabat. | | | | | | | | |
| --- | --- | --- | --- | --- | --- | --- | --- | --- |
| **V_H_** | | | |  | **V_L_** | | | |
| Residue | IMGT | Eu | Kabat |  | Residue | IMGT | Eu | Kabat |
| Q | 44 | 39 | 39 |  | Q | 44 | 38 | 38 |
|  |  |  |  |  |  |  |  |  |
| **C_H_1** | | | |  | **C_κ_** | | | |
| Residue | IMGT | Eu | Kabat |  | Residue | IMGT | Eu | Kabat |
| L | 7 | 128 | 124 |  | F | 5 | 116 | 116 |
| S | 13 | 134 | 130 |  | F | 7 | 118 | 118 |
| A | 20 | 141 | 139 |  | Q | 13 | 124 | 124 |
| G | 22 | 143 | 141 |  | T | 18 | 129 | 129 |
| L | 24 | 145 | 143 |  | S | 20 | 131 | 131 |
| K | 26 | 147 | 145 |  | V | 22 | 133 | 133 |
| D | 27 | 148 | 146 |  | L | 24 | 135 | 135 |
| Q | 84.2 | 175 | 179 |  | V | 82 | 163 | 163 |
| V | 88 | 185 | 190 |  | S | 85.1 | 174 | 174 |
| T | 90 | 187 | 192 |  | S | 86 | 176 | 176 |
|  |  |  |  |  | T | 88 | 178 | 178 |
| **C_H_3** | | | |  | T | 90 | 180 | 180 |
| Residue | IMGT | Eu | Kabat |  |  |  |  |  |
| L | 7 | 351 | 372 |  |  |  |  |  |
| T | 22 | 366 | 389 |  |  |  |  |  |
| L | 24 | 368 | 391 |  |  |  |  |  |
| P | 82 | 395 | 423 |  |  |  |  |  |
| F | 85.1 | 405 | 436 |  |  |  |  |  |
| Y | 86 | 407 | 438 |  |  |  |  |  |
| K | 88 | 409 | 440 |  |  |  |  |  |

1 10 15 16 23 26 27 38 39 41 45 77 84 85 89 96 97 104 105 117 118

87654321|........|....|123|......|..| |........| |.|...|1234567|......|12345677654321|...|......|12|......| |...........| |.........

C_H_3 GQPREPQVYTLPPSRDELT...KNQVSLTCLVK GFYP..SDIA VEWESNGQPEN...NYKTTPPVLDSD......GSFFLYSKLTVDKSRW..QQGNVFSC SVMHEA.LHNHYT QKSLSLSP

C_κ_ RTVAAPSVFIFPPSDEQLK...SGTASVVCLLN NFYP..REAK VQWKVDNALQSG..NSQESVTEQDSKD.....STYSLSSTLTLSKADY..EKHKVYAC EVTHQG..LSSPV TKSFNRGEC

C_H_1 ASTKGPSVFPLAPSSKSTS...GGTAALGCLVK DYFP..EPVT VSWNSGALTS....GVHTFPAVLQSS......GLYSLSSVVTVPSSSL...GTQTYIC NVNHKP..SNTKV DKKV

**Fig. 1-S**. IMGT sequence alignment (Lefran*c et a*l., 2015) of C_H_3 of IgG1, C_κ_ and C_H_1. Residues in C_H_3 previously chosen to induce C_H_3:C_H_3 repulsion (Yin*g et a*l., 2012) and their homologs in C_L_ are indicated in green, the conserved cysteine residues in yellow. Numbering is according to IMGT.
